# Biological Control of Phytopathogens: Mechanisms and Applications

**DOI:** 10.3390/pathogens12060783

**Published:** 2023-05-31

**Authors:** Anton Hartmann, Diogo Neves Proença

**Affiliations:** 1 Department of Biology, Microbe-Host Interactions, Ludwig-Maximilian-University München (LMU), Großhaderner Str. 2, 82152 Munich, Germany; 2 Department of Life Sciences, ARISE, CEMMPRE, University of Coimbra, Calçada Martim de Freitas, 3000-456 Coimbra, Portugal

## 1. Biocontrol Mechanisms

According to the inherent ecological mechanisms within community structures, organismic interactions are mediated by chemical structures and signaling molecules as well as enzymatic activities targeting the vital activities of microbial competitors. In addition, plants respond to microbial signals to build up defense against pathogen attack. Several contributions in this Special Issue present the latest information about the role of small diffusible molecules of rhizosphere microbes, fostering defense activities against pathogens. The review of Dias et al. [1] focuses on C4-bacterial volatile compounds (BVCs), such as 2,3-butanediol and acetoin, of plant growth-promoting rhizobacteria (PGPR), which are able to activate the induced systemic resistance of plants and promote plant growth. Other examples of diffusible molecules exerting interkingdom signaling effects on plants are quorum-sensing compounds of Gram-negative bacteria, e.g., *N*-acyl-homoserine lactones (AHLs). The opinion paper of Hartmann et al. [2] summarizes evidence that AHLs and AHL-producing bacteria induce efficient priming processes of pathogen defense and insect pest control. Furthermore, bacterial siderophores are known as important players for the competitive direct biological control of plant pathogens. Betoudji et al. (2020) present evidence that a siderophore analogue of Fimsbactin has antibacterial activities against *Pseudomonas syringae* pv. *tomato* DC3000 (*Pst* DC3000) and also confers resistance against *Pst* DC 3000 by inducing the systemic priming of resistance in *Arabidopsis thaliana* [3]. The antifungal activity of a cyclic tetrapeptide from *Bacillus velezensis* CE100 confers biological control against the plant pathogen *Colletotrichum gloeosporoides* by inhibiting mycelial growth and spore germination [4]. The root-endophytic fungus *Serendipita indica* (=*Piriformospora indica*) has the capacity to induce resistance in many host plants. In addition, Roylawar et al. (2021) report on the biocontrol activity of *S. indica* against onion leaf blight disease caused by *Stemphylium vesicarium* [5]. In particular, colonization with *S. indica* provides protection against peroxidative damage by inducing enzyme activities that protect against oxidative stress in onion plants. Finally, bacteria of the genus *Serratia* can contribute to the biological control of the pathogenic pinewood nematode (PWN) *Bursaphelenchus xylophilus*, which causes worldwide pine wilt disease. Marques-Pereira et al. (2022) studied the role of serratamolide-like amino lipids in the *Serratia–B. xylophilus* interaction and also researched *Caenorhabditis elegans* as a model organism [6]. The insights these studies provide will lead to a deeper understanding of the different biocontrol mechanisms of a wide variety of phytopathogens and open new possibilities for targeted and more effective applications.

## 2. Novel Biocontrol Bacteria

The biocontrol of pathogenic fungi-based Corm Rot disease of Saffron (*Crocus sativus* L.) was demonstrated by a novel rhizosphere actinomycete, *Streptomyces yangpuensis* CM253 [7]. The antagonistic actinomycete inhibits the growth and development of, e.g., *Fusarium solani*. Based on the analysis of degrading enzyme activities and the whole genome, numerous relevant genes and enzymatic activities were identified, which are involved in the biological control of Corm Rot. The growth and production of tomato and carrot are endangered by several bacterial and fungal pathogens that cause severe crop damage. Two plant growth-promoting actinomycete bacteria, *Streptomyces albidoflavus* H12 and *Nocardiopsis aegyptica* H14, were found to produce diffusible and volatile antifungal and antibacterial compounds [8]. Used as consortium, the infection with phytopathogens was reduced, improving the plant health status. Doty et al. (2023) report on the isolation of endophytic bacteria of poplar (*Populus*) belonging to the genera *Burkholderia*, *Bacillus* and *Pseudomonas*, having robust in vitro antifungal activities against agricultural pathogens such as *Rhizoctonia solani*, *Fusarium culmorum*, *Gaemannomyces graminis* and *Pythium ultimum* [9]. Genes for the synthesis of antibiotically active compounds were detected in these biocontrol active bacteria. Thus, the wild poplar tree microbiome could be a rich source of beneficial biocontrol bacteria.

## 3. Novel Detection Methods

The detection of phytoplasma that cause plant diseases is a most challenging task. Using a nested PCR-based detection approach [10], phytoplasma was reliably identified in leaf wilt disease of coconut in Sri Lanka. In Citrus plants, a quantitative PCR test is now available to detect the pathogen *Candidatus Liberibacter asiaticus*, a non-culturable, phloem-restricted bacterium causing Huanglongbing disease [11]. A biocontrol approach using the endophytic bacterium *B. subtilis* strain L11-21 was developed using quantitative PCR testing of the pathogen.

## 4. Applications

In recent years, research on the application of microbial biocontrol agents has made substantial progress in several ways. In China, there has been a steady and very substantial increase in the development and application of the biological control of phytopathogens, as reported by Meng et al. [12]. This is the result of China’s environmental protection policy in the last two decades, with increased attention on environmental protection, pollution control and ecological restoration. In other countries, colleagues also report on the increased application of sustainable biological approaches for plant growth promotion and pathogen control, which is based on the increased awareness and demand of the consumers. However, the progress cannot be documented as clearly as in the case of China. An alternative approach to chemical fumigation to control soil-borne pathogens could be anaerobic soil disinfection (ASD), as reported by Priyashantha et al. [13]. This involves the application of easily degradable carbon sources for the stimulation of soil activities followed by irrigation to field capacity and the maintenance of anaerobic conditions for some period. A quite unexpected way to improve plant health, as presented by Wassermann et al. [14], is based on the perception of natural and/or synthetic sound vibration (SV) by plants. A grapevine leaf-associated microbiota exposed to classical music was compared to non-exposed control leaves. Several host-beneficial, pathogen-antagonistic and even volatile organic-compound-producing bacteria were more abundant in SV-exposed phyllosphere populations. Finally, arbuscular mycorrhizal fungi (AMF) should be kept in mind as natural biofertilizer, biocontrol and bioprotective microbes in most agricultural crops. Bohacz et al. (2022) investigated AMF populations in spelt cultivars, a less studied “old” cereal [15].

## 5. Conclusions

In conclusion, the collected articles in this Special Issue certainly represent only a small subsample of the currently very productive research on biocontrol approaches of phytopathogens. More detailed knowledge about the different biocontrol mechanisms, which are sometimes even active synergistically in effective biocontrol microbes, will provide a more sound basis for successful practical applications in agriculture.
